# Synchronization of the circadian clock by time-restricted feeding with progressive increasing calorie intake. Resemblances and differences regarding a sustained hypocaloric restriction

**DOI:** 10.1038/s41598-020-66538-0

**Published:** 2020-06-22

**Authors:** Ana Cristina García-Gaytán, Manuel Miranda-Anaya, Isaías Turrubiate, Leonardo López-De Portugal, Guadalupe Nayeli Bocanegra-Botello, Amairani López-Islas, Mauricio Díaz-Muñoz, Isabel Méndez

**Affiliations:** 1 Instituto de Neurobiología, Universidad Nacional Autónoma de México (UNAM), Campus UNAM-Juriquilla, Querétaro, 76230 México; 2 Unidad Multidisciplinaria de Docencia e Investigación, Facultad de Ciencias, Universidad Nacional Autónoma de México (UNAM), Campus UNAM-Juriquilla, Querétaro, 76230 México

**Keywords:** Cell biology, Circadian rhythms, Carbohydrates, Hormones, Biochemistry, Lipids, Fatty acids, Physiology, Metabolism, Fat metabolism, Feeding behaviour, Homeostasis

## Abstract

Circadian rhythms are the product of the interaction of molecular clocks and environmental signals, such as light-dark cycles and eating-fasting cycles. Several studies have demonstrated that the circadian rhythm of peripheral clocks, and behavioural and metabolic mediators are re-synchronized in rodents fed under metabolic challenges, such as hyper- or hypocaloric diets and subjected to time-restricted feeding protocols. Despite the metabolic challenge, these approaches improve the metabolic status, raising the enquiry whether removing progressively the hypocaloric challenge in a  time-restricted feeding protocol leads to metabolic benefits by the synchronizing effect. To address this issue, we compared the effects of two time-restricted feeding protocols, one involved hypocaloric intake during the entire protocol (HCT) and the other implied a progressive intake accomplishing a normocaloric intake at the end of the protocol (NCT) on several behavioural, metabolic, and molecular rhythmic parameters. We observed that the food anticipatory activity (FAA) was driven and maintained in both HCT and NCT. Resynchronization of hepatic molecular clock, free fatty acids (FFAs), and FGF21 was elicited closely by HCT and NCT. We further observed that the fasting cycles involved in both protocols promoted ketone body production, preferentially beta-hydroxybutyrate in HCT, whereas acetoacetate was favoured in NCT before access to food. These findings demonstrate that time-restricted feeding does not require a sustained calorie restriction for promoting and maintaining the synchronization of the metabolic and behavioural circadian clock, and suggest that metabolic modulators, such as FFAs and FGF21, could contribute to FAA expression.

## Introduction

The circadian timing system acts as a physiological coordinator from the molecular to the organismic level. This system consists of an organised set of daily central and peripheral oscillators (with the suprachiasmatic nucleus as principal pacemaker) whose activity is tuned in to endogenous influences (molecular circadian clock, redox state, metabolic networks) and synchronized by various environmental factors (photic stimulation, food access, social interaction)^1^. These rhythms are indispensable for metabolic homeostasis, and their disruption is involved in the onset and development of several pathologies including obesity, dyslipidaemia, liver diseases, diabetes, and cancer^2–5^.

Food availability can act as a prevailing timing cue for circadian synchronization, even in conditions of a non-functional suprachiasmatic nucleus, indicating the existence of an alternative physiological strategy to measure biological time, independent of photic stimulation^6^. This timing system, known as the food entrainable oscillator (FEO), is activated in response to a variety of time-caloric restriction protocols in which food access is limited to a few hours per day^7–12^. Indeed, the dual relationship between the time-recording molecular circadian clock and the metabolic networks that sustain the cellular energetic reactions have key impact on the molecular establishment of FEO^13^. Calorie restriction, the decrease of calorie intake by up to 40%, is a potent stimulus that delays the onset of multiple age-associated diseases and improves metabolic health by reducing body weight and total fat depots^14,15^. The reduced caloric intake in restricted feeding schedules elicits a phase shift in molecular and metabolic machinery components in peripheral clocks, including the liver^16,17^. Likewise, imposed periods of extended daily fasting, independent of dietary composition and calorie intake, have significant metabolic and lifespan benefits^18,19^. However, metabolic improvements have been observed in mice fed only with high-fat diet under restricted feeding schedules^20,21^, and it is still unknown whether a normocaloric diet is able to confer the metabolic benefits of time-restricted feeding (TRF) involving circadian clock synchronization. In addition, a hallmark associated with TRF and the FEO expression is the appearance of a distinctive outbreak of locomotor activity preceding the presentation of food, known as food-anticipatory activity (FAA)^7^. Daytime restricted feeding (2 h of food access) in rats is a hypocaloric condition (HCT) that elicits the expression of FAA as the behavioural output of the FEO^22^. Based on this information, the present study aimed at elucidating whether a protocol of daytime restricted feeding that progressively accomplishes a normocaloric intake (NCT), using a standard lab rodent diet, was able to induce metabolic signals associated to FAA. To achieve this objective, we extended the feeding time for rats in the restricted protocol to 5 h of food access to allow circadian food synchronization. We observed that restricting the timing of food intake during 21 days promoted a sustained expression of the FAA, and, at the end of the protocols, it induced the synchronization of hepatic clock genes, and the elevation of circulating free fatty acids (FFAs), regardless of calorie intake during the protocols. In contrast, parameters such as endocrine responses, adipose tissue amount and morphology, hepatic pro-oxidant reactions, redox regulation, and oxidised or reduced forms of ketone bodies, varied according to the ingested calorie intake. Overall, these data allow us to further understand the molecular and energetic elements that underlie circadian synchronization by restricted feeding schedules and the associated FEO expression.

## Results

### Models of  time-restricted feeding with a sustained hypocaloric intake (HCT) and a progressive increasing calorie intake (NCT)

**Figure 1:**
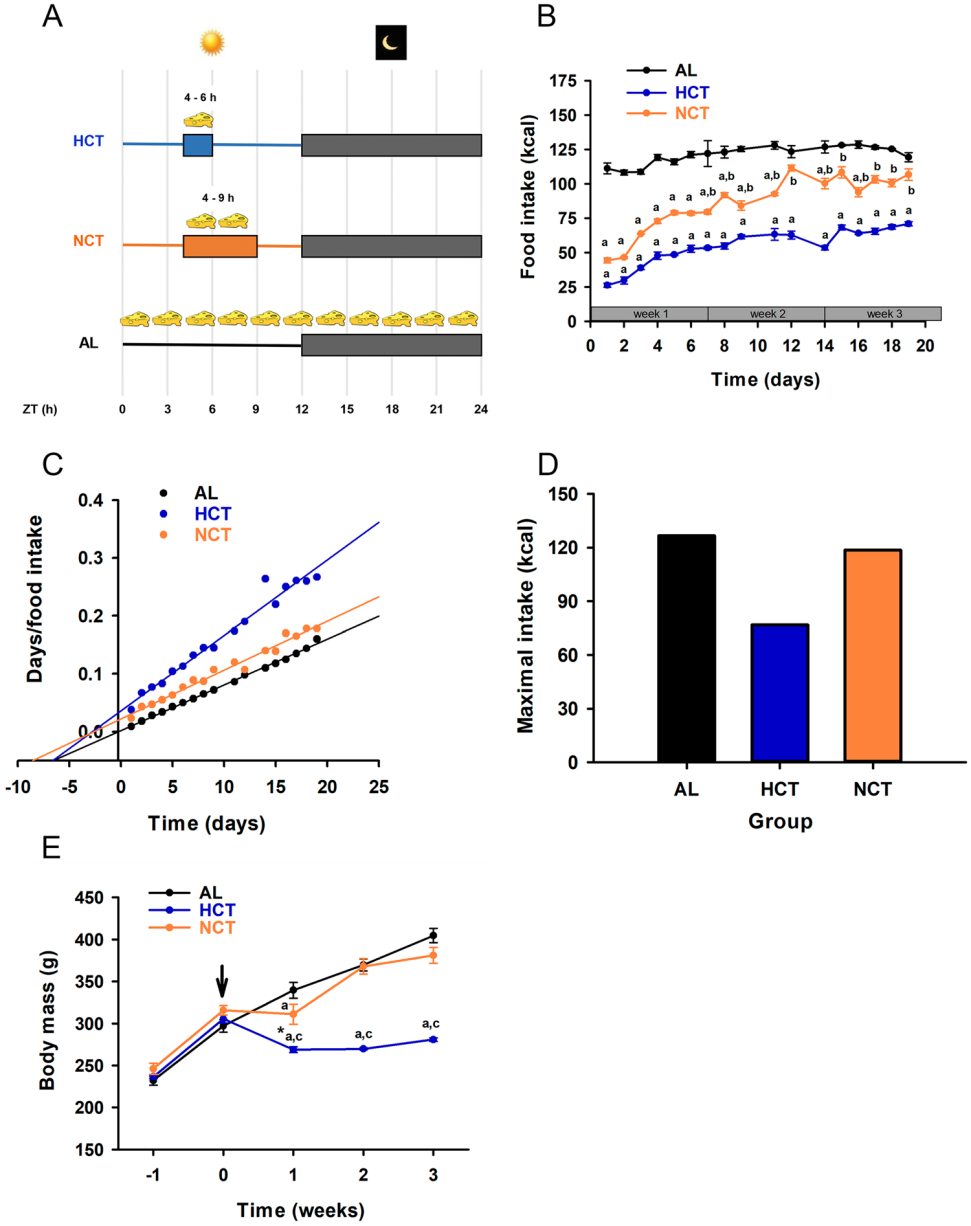
Characteristics of food intake and weight gain in progressive increasing to normocaloric intake (NCT) and sustained hypocaloric intake (HCT) protocols of time-restricted feeding. (**A**) Schematic of the experimental protocols indicating time window of food access during a 24 h day under *ad libitum* feeding (AL, n = 12 rats), HCT (food access for 2 h, n = 10 rats) and NCT (food access for 5 h, n = 10 rats). (**B**) Food intake during 3 weeks of the experiment. Each point represents the mean ± SEM. *P* < 0.05 a vs AL, b vs HCT. (**C**) Analysis of food intake curves by Hanes–Woolf method. (**D**) Maximal food intake according to lineal normalisation. (**E**) Weekly weight gain during 3 weeks of the experiment; row indicates the beginning of the protocols. *P* < 0.01 *vs week 0 in HCT, a vs AL, c vs NCT.

To compare the effect of both protocols of calorie intake on metabolic and molecular rhythm parameters, groups of rats were fed normal chow *ad libitum* (AL) or subjected to daily fasting-refeeding cycles with access to food either for 2 h (ZT4-ZT6, HCT) or 5 h (ZT4-ZT9, NCT) during 3 weeks (Fig. 1A). The daily food intake is represented in Fig. 1B. Rats fed AL showed a stable pattern of ingested food throughout the protocol. The HCT group showed a pattern that started with a significant reduction of the calorie intake comparing to AL condition and reached a plateau at the end of the protocol (45% calorie restriction); therefore, this involves hypocaloric intake as previously reported^16^. While the NCT group started with a 40% calorie restriction in the first week, it reached 90% of the calorie intake of AL rats at week 3. Curves of food intake were subjected to lineal analysis according to the Hanes–Woolf method; in this representation, the slope indicates the maximum food intake, whereas the x-intercept is an index of half the time to reach the maximum food intake (Fig. 1C). The maximum food intake for the AL and NCT groups was similar (127.4 and 118.6 kcal, respectively), whereas for the HCT group it was clearly reduced (76.8 kcal) (Fig. 1D). The normalised data indicated a discrete 7% reduction in the NCT group and a 40% reduction in the HCT group (Supplementary Table S2). Body weight was measured weekly throughout the experiment (Fig. 1E). During the first week of adaptation, the 3 groups of rats gained weight in a similar way. From the beginning, HCT rats diminished their body weight by 12% and maintained it for the rest of the protocol. In contrast, AL and NCT groups gained weight in a similar fashion, mainly from week 2, indicating that 5 h of food access provided the NCT group with the necessary calories to promote normal growth.

To corroborate that the HCT protocol involves a sustained calorie restriction but not malnutrition, we measured serum total protein and found no differences between the three feeding conditions after 21 days (Supplementary Fig. S1A). Moreover, the sustained calorie restriction did not affect the serum lactate dehydrogenase activity (Supplementary Fig. S1B) which, according to a previous report is elevated under chronic calorie restriction^23^. The metabolic outcomes of HCT and NCT protocols were evaluated according to the performance in metabolic cages and compared to AL. The respiratory exchange ratio and respiratory quotient (RQ) rhythms showed nocturnal acrophases in the AL condition, and a marked phase-shift from nocturnal to diurnal increase in both, HCT and NCT conditions (Supplementary Fig. S1C). The rhythm in energy expenditure substantially increased during the dark phase and decreased during the light phase under AL condition. A phase shift- was observed in this rhythm under both, HCT and NCT conditions, with a peak during the day, which was consistent with the time of feeding and then dropped during the dark phase. (Supplementary Fig. S1D). These data indicate that TRF, regardless of calorie intake, elicits a similar time shift in the utilisation of substrates and energy expenditure compared to AL.

### Food anticipatory activity arises in HCT and NCT

**Figure 2:**
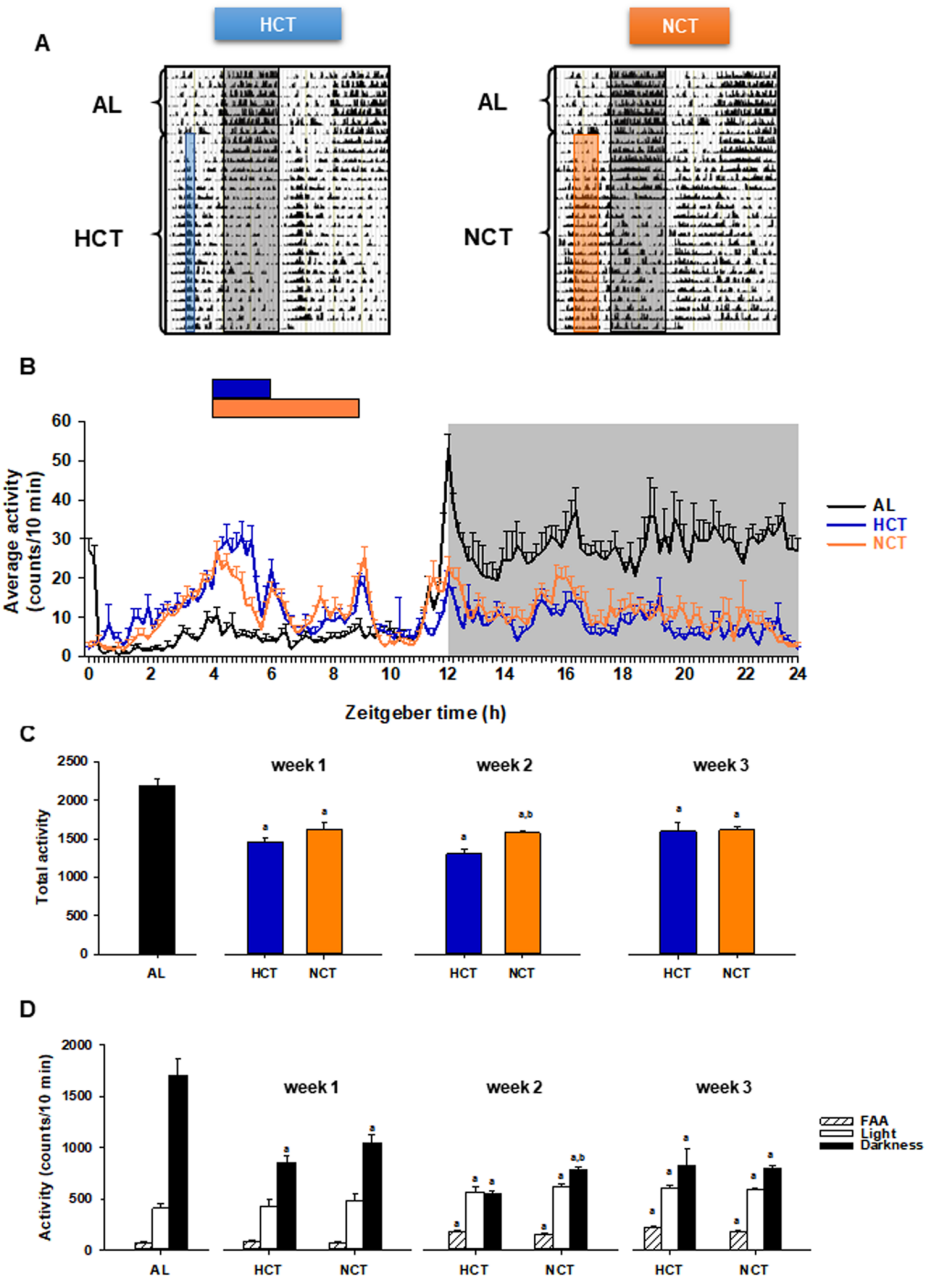
Locomotor activity under AL n = 10 rats), HCT (n = 10 rats) and NCT (n = 10 rats) protocols of  time-restricted feeding in 12:12 h light-dark cycles. (**A**) Representative double-plotted actograms showing the daily locomotor activity of rats. Each horizontal line represents 2 days  of recording. Both groups were kept in individual cages for 1 week under AL and then during 3 weeks under the respective time-restricted feeding protocol; food access is represented by vertical bars (blue, HCT; orange NCT) and darkness by the grey bar.(**B**) Activity profiles of rats over 24 h cycle. Waveforms represent the sum into 10-min bins and average of 5 days of recording under AL, HCT or NCT. Horizontal bars represent the time to food access (blue, HCT; orange NCT). Each point represents the mean ± SEM. (**C**) The average of the total activity under AL conditions and throughout each of the 3 weeks under the respective protocol. *P* ≤ 0.001 a vs AL, b vs HCT. (**D**) The representation of 3 h before food access (ZT1-ZT4) corresponding to spontaneous basal activity in AL condition (grey bar) and the food-anticipatory activity (FAA) in HCT and NCT (dashed bars), the rest of the light period (ZT4–ZT12) and the darkness (ZT12–ZT24) and throughout the 3 weeks of the respective protocol. Bars represent the mean ± SEM. *P* ≤ 0.001 a vs AL, b vs HCT.

When food is provided daily at a fixed time of day, physiological anticipation occurs in animals to obtain the maximal benefit. Under TRF protocols, rats exhibited daily rhythms of food anticipatory activity (FAA) during the 2–3 h preceding food access, which is considered the outcome of an entrainment process by feeding based on the coordinated control of peripheral and central circadian clocks^24^. FAA magnitude and onset in mice is directly related to the degree of caloric restriction during the TRF protocol^25^. In order to know if a sustained hypocaloric intake is needed to express the FAA induced by temporal restricted feeding in rats, our initial approach was to investigate whether FAA was present by measuring and comparing locomotor activity under NCT and HCT. After 1 week of acclimation, rats AL were monitored for 1 week and then subjected either to HCT or NCT for 3 weeks. The AL condition showed a typical nocturnal pattern of activity with few bouts during the day (Fig. 2A). In contrast, we observed a FAA component in both, HCT and NCT groups 3 h before the onset of food access (ZT1-ZT4) since the fourth day of restriction (Fig. 2A,B). These data were corroborated using an area under the curve plot (Supplementary Fig. S2) and with the sum of activity during FAA, which significant augmented as the time elapsed (Fig. 2D). Compared to the AL group, the total activity was reduced by nearly 30% by both, HCT and NCT (Fig. 2C), the amplitude of activity was reduced during the night and spread throughout the day, as seen in the average waveform corresponding to the third week of the restriction protocols (Fig. 2B). Remarkably, under continuous darkness and fasting conditions, FAA persisted in both food-synchronized groups at the same magnitude, as well as the total locomotor activity (Supplementary Fig. S2B,C), independently of calorie intake during restriction protocols.

### A similar phase shift of clock genes was induced by HCT and NCT

**Figure 3:**
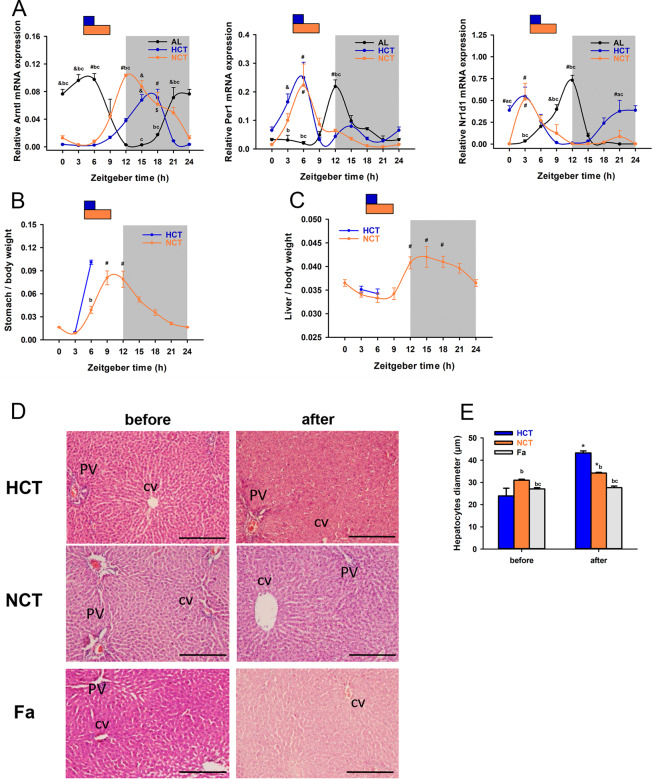
Circadian profile of liver clock genes and metabolic organs weight under HCT and NCT at day 21 of each protocol (n = 4 rats). (**A**) Relative mRNA expression of Bmal1 (Arntl), Per1 (Per1) and Rev-erb-α (Nr1d1) in liver analysed by qPCR and normalised to Rps18. Food access is represented by vertical bars (blue, HCT; orange NCT) and darkness by the grey bar. Each point represents the mean ± SEM. *P* < 0.05 a vs AL, b vs HCT, c vs NCT. *P* < 0.05 # vs ZT0, 3, 6, 9, 18, 21; $ vs ZT3, 6, *P* < 0.01 & vs ZT0, 3, 6, 21 in NCT and HCT. *P* < 0.01 # vs ZT9, 12, 15, 18; & vs ZT12, 15 in AL. (**B**) Relative stomach weight and (**C**) liver weight normalised to body weight in HCT and NCT. *P* < 0.01 # vs ZT0, 3 in stomach weight and vs ZT3, 6, 9 in liver weight; *P* < 0.05 b vs HCT. (**D**) H&E histological sections of representative livers under HCT, NCT and single fasting for 21 h (Fa) before (ZT3) and after (ZT6 for HCT and Fa or ZT9 for NCT) feeding. Venous references are indicated as CV (central vein) and PV (portal vein) (scale bar= 500 μm). (**E**) Hepatocyte sizes measured in 3 fields per tissue section from 3 rats of each group. Bars represent the mean ± SEM. *P* < 0.01 b vs HCT, c vs NCT. *P* < 0.01 * vs before food access in their respective group.

Nutritional challenges, such as a high-fat diet or calorie restriction, elicit the reprogramming of the molecular circadian clock of peripheral organs without affecting the central circadian clock^26,27^. The liver plays a central role in metabolic actions, and its circadian rhythmicity is highly responsive to feeding protocols. To measure the impact of the NCT compared to HCT, we quantified the daily pattern of core clock genes Per1 and Bmal1, and the accessory gene Rev-Erb-α over a 24 h cycle in the liver. Both TRF protocols closely shifted the phase of the mRNA expression independently of the calorie intake (Fig. 3A and Table 1), except for the acrophase of Rev-Erb α, which advanced 4 h in HCT with respect to NCT.

**Table 1:**
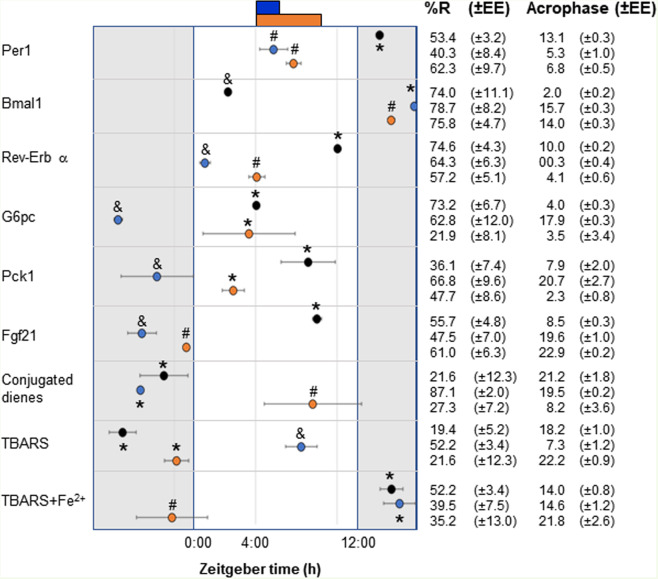
Chronobiological analysis of clock, metabolic and redox state in liver under AL, HCT and NCT.

Windows of time of food access (blue, HCT; orange, NCT), light phase (white bar), and dark phase (grey bar) are represented. Different symbols represent statistical differences among acrophases by one-way ANOVA with a Tukey post-hoc test (*P *< 0.01).

### Differential changes in metabolic organs induced by HCT and NCT

Previously, it was shown that HCT provoked sudden stomach distension after food access (ZT3) with an immediate but gradual emptying until the time just before the next food access^16^. We observed a different effect in the NCT protocol: a smaller increase in stomach distension and a similar emptying rate of the stomach content until the time before food access (Fig. 3B). Regarding liver weight, we did not observe significant differences associated with NCT during a 24 h cycle (Fig. 3C), compared to the changes previously reported in the HCT protocol^16^. However, the histological analysis showed different characteristics between the tissue architecture of NCT and HCT groups before (ZT3) and after (ZT6 for HCT and ZT9 for NCT) food access (Fig. 3D). Both groups differed significantly regarding hepatocyte cell sizes and compared to a single 21 h fasting group (before, ZT3 and after, ZT6) (Fig. 3E). Sinusoids were observed more evident, especially before food access, regardless of the feeding protocol; and they rapidly hid only under the HCT condition probably because of the space occupied by the significant increment in hepatocyte size after refeeding (Fig. 3E). Given that fasting conditions decrease hepatocyte volume, possible due to a decrease in glycogen and protein content, these data suggest that hepatocytes from the NCT condition could be metabolically different to HCT before and after feeding, and that influence of restricted feeding on cells size is different from a single fasting.

### Similar hypoglycaemic states but different gluconeogenic, glycogenolytic, and insulin responses promoted by HCT and NCT

**Figure 4:**
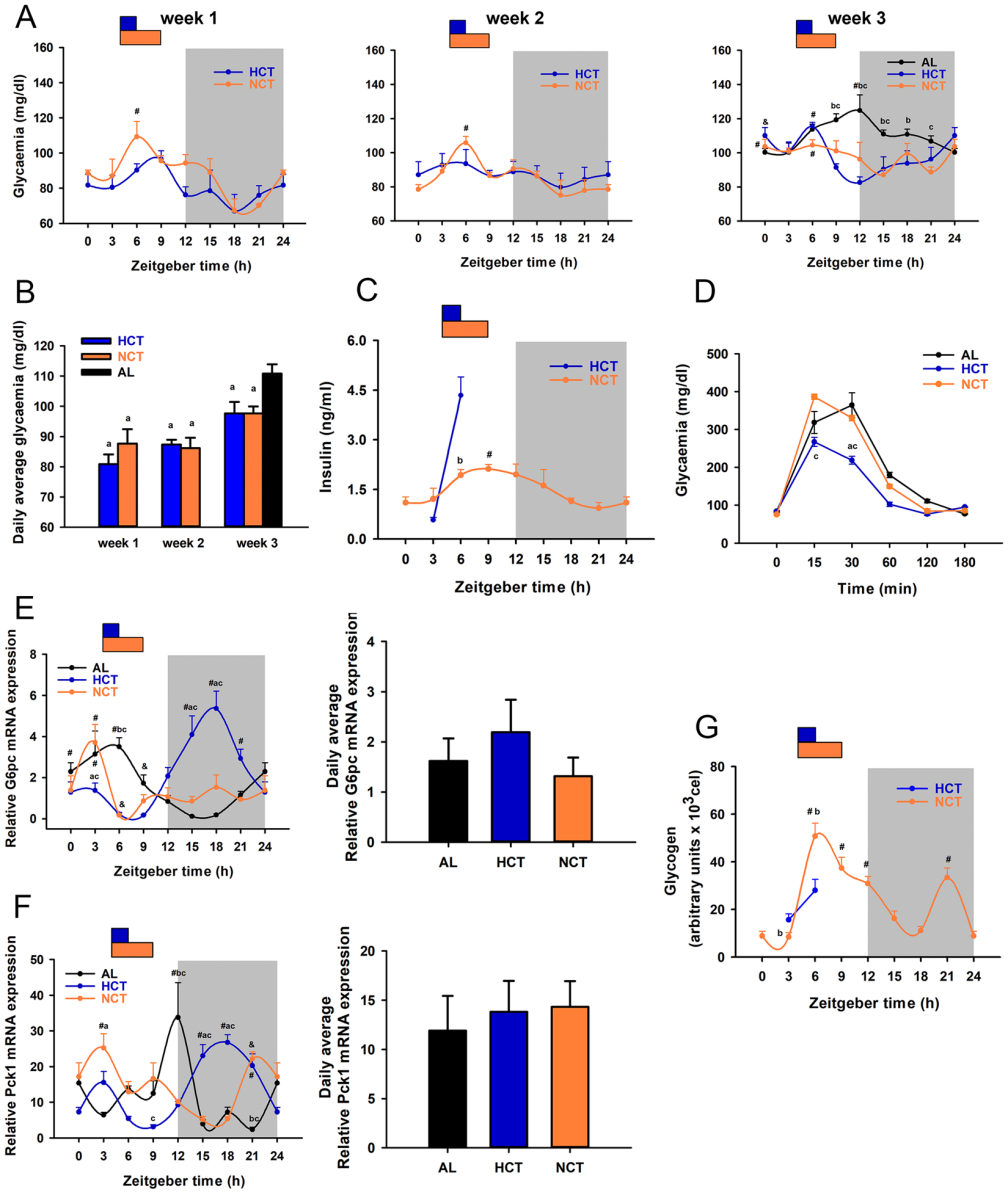
Effect of HCT and NCT on glucose metabolism (n = 4 rats). (**A**) Daily profile of glycaemia during the 3 weeks of the respective protocol. AL daily profile is shown in the week 3. Each point represents the mean ± SEM. In week 1 and week 2, *P* < 0.05 # vs ZT18, 21; in week 3, *P* < 0.05 # vs ZT0, 3 in AL; vs ZT15, 21 in NCT; vs ZT9, 12, 15, 18 and *P* < 0.01 & vs ZT12 in HCT; *P* < 0.05 b vs HCT, c vs NCT. (**B**) Daily average glycaemia during the 3 weeks of each protocol. *P* < 0.05 a vs AL. (**C**) Daily profile of serum insulin concentrations under NCT and before and after food access under HCT. *P* < 0.05 # vs ZT21; *P* < 0.01 b vs HCT. (**D**) Glucose tolerance test. *P* < 0.05 a vs AL, c vs NCT. (E-F left) Daily profile of the mRNA expression of (**E**) glucose-6-phosphatase (G6pc). *P* < 0.05 a vs AL, b vs HCT, c vs NCT. *P* < 0.05 # vs ZT6, 9 in HCT and NCT, & vs ZT3, 18, 21 in NCT; *P* < 0.01 & ZT15 in AL; *P* < 0.01 # vs ZT9, 12, 15, 18; & vs ZT12, 15 in AL. (**F**) Phosphoenolpyruvate carboxykinase (*P*ck1). *P* < 0.01 a vs AL, b vs HCT, *P* < 0.05 c vs NCT. *P* < 0.05 # vs ZT0, 6, 9, 12 in HCT, *P* < 0.05 # vs ZT15, 18 in NCT, *P* < 0.01 # vs ZT0, 3, 6, 9, 15, 18, 21 in AL. (E-F right). Daily average of mRNA expression. Bars represent the mean ± SEM. (**G**) Content of liver glycogen evaluated by PAS staining during a 24 h period under NCT and before and after food access under HCT. *P* < 0.01 b vs HCT. *P* < 0.001 # vs ZT0, 3, 15, 18 in NCT.

Glycaemia displays clock gene-dependent oscillations during a 24 h cycle with a peak during the nocturnal meal-time^28^, whereas 2 h food restriction in the rest phase for 3 weeks (HCT) promotes changes in this rhythmicity pattern and hypoglycaemia^9^. To analyse the effect over the glycaemia, we evaluated the changes throughout the 3 weeks and observed gradual changes along the day in each group (Fig. 4A). From the first week, both restricted feeding conditions induced a hypoglycaemic state, but we observed a rhythmic pattern only in NCT (Fig. 4A). At week 3, we observed a shift phase comparing to AL condition with the peak of glycaemia at ZT6 in both, HCT and NCT conditions. The sustained hypocaloric intake (HCT) led the peak of glycaemia to rapidly drop, while in NCT glycaemia slowly diminished, and remained low throughout the rest of the day, presenting another peak just at the beginning of the light period (ZT0). It should be noted that daily average glucose concentration decreased significantly from the first week by both restriction protocols, regardless of the calorie intake (Fig. 4B). Hypoglycaemia could be associated with putative endocrine responses after food consumption; however, although the calorie intake was significantly lower in HCT, the glucose level was similar in HCT and NCT during the 3 weeks of the experiment.

Regarding the insulin response associated with the hypoglycaemic action over both restricted feeding protocols, the 24 h insulin profile under the NCT condition differed significantly from the insulin profile under HCT, as previously reported^9^. Before food access (ZT3), insulin levels were similar in both protocols. At ZT6, however, a damped insulin profile was observed in NCT unlike the prominent peak present in HCT (Fig. 4C). Besides, the glucose tolerance test showed similar patterns in AL and NCT, whereas a more sensitive response to insulin was detected in the HCT group probably due to the peak of insulin (Fig. 4D). The present data suggest that HCT and NCT improve circulating glucose levels through a different insulin-dependent mechanism.

To maintain glycaemia, prolonged fasting induces hepatic gluconeogenesis, a biochemical pathway that is transcriptionally repressed by food intake and insulin. Rhythmic expression of gluconeogenic genes glucose-6-phosphatase (G6pc) and phosphoenolpyruvate carboxykinase (Pck1) was analysed to determine the adaptative changes in glucose handling to fasting-refeeding cycles in HCT and NCT. Compared with AL, temporal restricted feeding promoted a phase shift of both G6pc and Pck1, without modifying the daily average mRNA expression (Fig. 4E,F and Table 1). The sustained hypocaloric intake in HCT generated a peak in the expression of both gluconeogenic genes during the dark period, while NCT induced a peak during the light period, previous to food access (Table 1). Interestingly, in NCT, corticosterone levels did not increase before the food access (Supplementary Fig. 2D), unlike in the sustained hypocaloric protocol in which elevation of circulating corticosterone during the time of FAA is considered as a hallmark of FEO expression^9,17^.

The availability of circulating glucose during fasting also depends on storages like liver glycogen depots. Thus, we evaluated the daily fluctuations of liver glycogen under the NCT protocol. Unlike HCT, which has been reported to present phase-shifting with a slow increase after refeeding and then a sudden drop with no total depletion of glycogen^9^, NCT promoted glycogen synthesis promptly after the onset of feeding, and a gradual reduction and total depletion of glycogen (Fig. 4G). Partial replenishment during the dark phase was present in both TRF protocols as a temporal adaptation, probably to ensure an energy store.

### Changes in the morphology of different adipose tissue depots in HCT without differences in function compared to NCT

**Figure 5:**
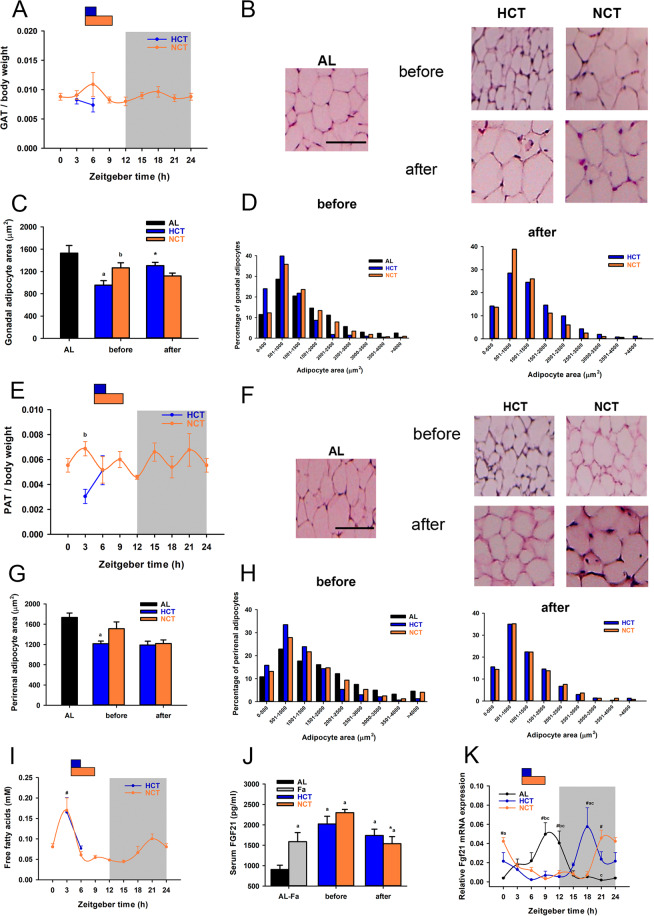
Effects of HCT and NCT on the structural and functional characteristics of adipose tissue at day 21 of each protocol. Daily profile of (**A**) gonadal (GAT) and (**E**) perirenal (PAT) adipose tissue weight normalised to body weight in NCT, and before and after feeding in HCT (n = 4 rats). Each point represents the mean ± SEM. *P* < 0.001 b vs HCT. The representative formalin-fixed paraffin-embedded H&E staining images of (**B**) gonadal and (**F**) perirenal adipose tissue from the different groups. Scale bars = 100 μm. Adipocyte surface area in (**C**) gonadal and (**G**) perirenal adipose tissue depots before (ZT3) and after (ZT6 for HCT or ZT9 for NCT) feeding. The area measured in approximately 300 cells per rat (4 rats total) was grouped in each graph. Bars represent the mean ± SEM. *P* < 0.05 a vs AL, b vs HCT, * vs before feeding in the same group. Bar charts of frequency distribution of (**D**) gonadal and (**H**) perirenal adipocyte cell area from AL, HCT, and NCT rats, before and after feeding. (**I**) Daily profile of FFAs measured in serum under NCT and before and after feeding under HCT. *P* < 0.001 # vs ZT15 in NCT. (**J**) Serum FGF21 levels in AL, single fasting for 21 h (Fa) and before and after feeding. *P* < 0.01 a vs AL, *P* < 0.01 * vs before food access in its respective group. (**K**) Daily profile of relative mRNA expression of Fgf21 in liver. *P* < 0.05 b vs HCT, c vs NCT; # vs ZT9, 12, 15, 18, # vs ZT3, 6, 9, 12 in HCT; # vs ZT0, 15, 18, 21 in AL.

Fasting and refeeding cycles involve rhythmic mobilisation of energy depots, such as white adipose tissue, to contend with nutritional and energetic requirements. Therefore, to learn more on the effects of NCT and HCT, we evaluated the structure and function of gonadal and perirenal adipose tissues. The temporal profile of both adipose tissues weight normalised to body weight showed non-significant changes during the 24 h cycle in NCT (Fig. 5A,E). Comparing both TRF groups, the gonadal adipose tissue weight in HCT was like NCT both, before (ZT3) and after feeding (ZT6 for HCT and ZT9 for NCT) (Fig. 5A). However, before feeding, the adipocyte area in HCT was reduced compared to NCT, and similar in both groups after refeeding (Fig. 5C). The frequency of small adipocytes was greater in HCT than in NCT and AL at ZT3, while the frequency of size shifted to larger adipocytes at ZT6 (Fig. 5B,D). By contrast, the perirenal adipose tissue weight was lower in HCT than in NCT before feeding and similar after the meal. The difference was reflected in the mean surface area of adipocytes (Fig. 5G). The frequency of small adipocytes in HCT was greater than in NCT and AL at ZT3, and adipocytes increased in size after feeding (ZT6 for HCT and ZT9 for NCT), being as large as in NCT (Fig. 5F,H). A diminished adipocyte area of both white adipose tissues in HCT comparing to NCT and AL at ZT3 reveals an extensive utilization of fat stores in a sustained hypocaloric intake. Thus, we observed some structural differences between two white adipose tissue depots in response to cycles of fasting and refeeding when comparing NCT and HCT protocols.

Based on these findings, we quantified FFAs in a temporal 24 h cycle. Before feeding (ZT3), we identified a peak of FFAs in response to fasting under HCT^8,10^. Despite the difference in caloric intake, we observed the same response under NCT (Fig. 5I), indicating a similar lipolytic activity by the adipose tissue under both protocols, independent of the changes in adipocytic structural characteristics induced by calorie intake.

Fibroblast growth factor 21 (FGF21) is an endocrine/paracrine factor that is part of the fasting response to mediate energy homeostasis regulating liver gluconeogenesis and β-oxidation^29^, and adipose tissue lipolysis^30^ and it acts centrally in brain areas involved in circadian behaviour during starvation^31^. Although Fgf21 mRNA is expressed in multiple metabolic sites, circulating FGF21 is mostly released by the liver^32^. Serum FGF21 levels increased in response to repeated fasting under NCT and HCT, as well as with a single 21 h fasting, and tended to diminish after refeeding (Fig. 5J). Fgf21 mRNA expression in liver displayed a 24 h rhythm in AL conditions and both restricted feeding protocols promoted a phase shift, independently of the calorie intake (Fig. 5K and Table 1). Thus, FGF21 could be a peripheral signal to increase energy expenditure and regulate fatty acid oxidation and ketogenesis in liver metabolism.

### Differential redox state and pro-oxidant reactions in liver induced by HCT and NCT

**Figure 6:**
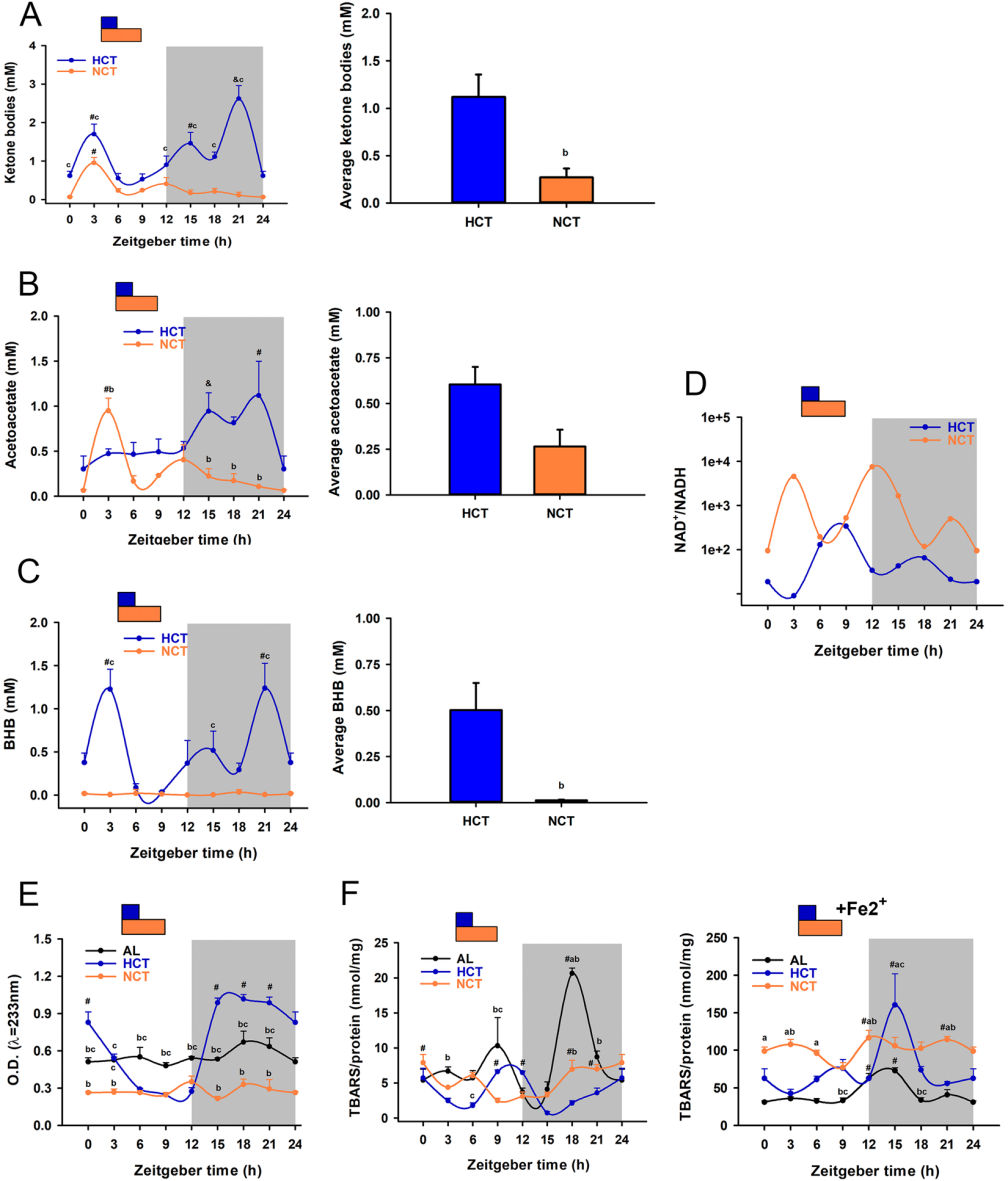
Effects of HCT and NCT on liver ketone bodies and redox status at day 21 of each protocol (n = 4 rats). Daily profile and daily average of (**A**) total serum ketone bodies, (**B**) acetoacetate and (**C**) β-hydroxybutyrate (BHB). (A-C left) Daily profile (A–C right) and daily average of serum ketone bodies. Each point represents the mean ± SEM. For total ketone bodies: *P* < 0.01 c vs NCT, # vs ZT0, 6, 15, 18, 21 in HCT, $ vs ZT0, 6, 9 in HCT; *P* < 0.05 & vs ZT0, 3, 6, 9, 12, 15, 18 in NCT; for acetoacetate: *P* < 0.01 b vs HCT, # vs ZT0, 6, 9, 15, 18, 21 in NCT; *P* < 0.05 # vs ZT0, 3, 6, 9 HCT, & vs ZT0 in HCT; for β-hydroxybutyrate: *P* < 0.01 c vs NCT, # vs ZT0, 6, 9, 12, 15, 18 in HCT. Bars represent the mean ± SEM. *P* < 0.05 b vs HCT. (**D**) Total redox state in terms of the NAD^+^/NADH ratio. Daily profile of lipoperoxidation in terms of (**E**) conjugated dienes, (F-left) TBARs and (F-right) TBARs + Fe^2+^. For conjugated dienes: *P* < 0.001 b vs HCT, c vs NCT; # vs ZT3, 6, 9, 12 in HCT. For TBARs: *P* < 0.05 a vs AL, b vs HCT, c vs NCT; # vs ZT9, 12, 15 in NCT, vs ZT15 in HCT, vs ZT12 in AL; for TBARs + Fe^2+^: *P* < 0.05 a vs AL, b vs HCT, c vs NCT; # vs ZT9 in NCT; vs ZT3 in HCT; vs ZT0, 3, 6, 9, 18, 21 in AL.

Ketone bodies, acetoacetate (oxidised form) and β-hydroxybutyrate (reduced form) are direct products of fatty acids β-oxidation within peroxisomes and mitochondria as a physiological response of fasting and are indicators of the mitochondrial redox state of the liver^33^. The acetoacetate/β-hydroxybutyrate ratio depends on the NAD^+^/NADH ratio present in the liver mitochondria; if NADH concentration is high, the liver will release a higher proportion of β-hydroxybutyrate. In this context, a high β-hydroxybutyrate/acetoacetate ratio is a marker of a reduced mitochondrial redox state, whereas a high acetoacetate/β-hydroxybutyrate ratio is indicative of an oxidised mitochondrial redox state. As control of ketone bodies production, we analysed them in the serum of rats under fasting (21 h), which induced 7-times more total ketone bodies than TRF and was restored by refeeding (Supplementary Fig. S3). NCT and HCT groups showed a peak of total ketone bodies at ZT3 (Fig. 6A), that is coincident with the peak of FFAs (Fig. 5I), with the remarkable finding of the differential redox ratio. This peak is dependent on the production of β-hydroxybutyrate in NCT and acetoacetate in HCT (Fig. 6B,C). Only HCT showed another peak at ZT21 (Fig. 6A) reliant on both, β-hydroxybutyrate and acetoacetate (Fig. 6B,C). In NCT, the levels of β-hydroxybutyrate showed a different pattern in comparison to HCT, being significantly lower and without rhythm during the 24 h cycle (Fig. 6C). Besides, the HCT group showed a significant elevation of average circulating ketone bodies, in comparison with the NCT group (Fig. 6A–C), suggesting an augmented fatty acid β-oxidation induced by the sustained hypocaloric intake. Mitochondrial redox analysis indicated that HCT displayed a reduced state before feeding and a discrete oxidised state after feeding (Fig. 6D). In contrast, NCT displayed an overwhelming oxidised state, especially in the light period and at the beginning of the dark period.

Generation of reactive oxygen species is important not only in conditions of clear oxidative stress but also as a part of the redox homeostasis that commands a set of different biochemical reactions involving electron flux towards oxygen. Thus, the redox state is one of the principal regulatory parameters for the control of metabolic networks, as electron flux dictates the balance between anabolic and catabolic reactions. Lipid peroxidation is a set of propagative reactions that take place mainly within biological membranes. Used as markers of lipid peroxidation, levels of conjugated dienes measured in liver homogenate did not show any fluctuation during the 24 h cycle under NCT and AL conditions (Fig. 6E). In contrast, under HCT, conjugated dienes presented a robust rhythmicity (Fig. 6E, Table 1) with levels 4-times higher during the dark period compared to the lowest levels during the light period, similar to the ones observed with the NCT group during the 24 h cycle (Fig. 6E).

Thiobarbituric acid reactive substances (TBARs) are considered as marker of the “pro-oxidant potential” of cells. The 24 h patterns of liver TBARs were different from the daily fluctuations of conjugated dienes. Both, NCT and HCT showed rhythmicity patterns during the 24 h cycle with reduced levels in comparison to AL (Fig. 6F and Table 1). Upon addition of Fe^2+^, a promoter of the Fenton reaction, TBARs levels were increased substantially (Fig. 6F). All groups presented rhythmicity patterns with differences in acrophase and TBARs levels (Fig. 6F and Table 1). NCT promoted a clear increment in TBARs values and a phase shift in comparison to AL and HCT groups, while HCT showed the most robust rhythm. Therefore, pro-oxidant reactions changed according to the caloric intake under temporal restricted feeding.

## Discussion

Time-restricted food access or  time-restricted feeding (TRF) with a sustained hypocaloric intake (HCT) consists in limiting the daily period of food intake to a short window of time (2 h of food access), which involves daily fasting-refeeding cycles, features that promote adaptations in metabolic and health indices by the synchronising effect of TRF. However, it is unclear whether these physiological responses are mediated by the metabolic challenge of the continuous calorie restriction during the protocol. In the present study, we found that TRF with a gradual increase of calories to achieve a normocaloric intake (NCT) elicits the metabolic adaptations associated with the feeding entrainment clock in young adult male rats under a protocol of 5 h of access to food, without the metabolic challenge of hyper or hypocaloric diets. Moreover, we found that the food anticipatory activity (FAA) is elicited by synchronization through TRF and can be mediated by additional metabolites to those dependent on β-hydroxybutyrate according to calorie intake.

Adjustment of caloric intake and meal frequency, specific periods of fasting and refeeding, have been demonstrated as powerful strategies to improve health^18,34,35^, even in the presence of a metabolic challenge involving a high-fat diet, by driving rhythms of key metabolic regulators of nutrient homeostasis such as CREB and AKT^34^. Furthermore, in the absence of a hypercaloric challenge (obesogenic diets), most TRF studies in rats restricted caloric intake by 20–40% during the entire protocol^16^, raising the question of whether removing progressively the hypocaloric challenge leads to metabolism adaptations by the synchronizing effect.

Calorie restriction has a profound impact on age-related disorders, for example, cancer and neurodegenerative and cardiovascular diseases^36^, and it diminishes metabolic indices, as insulin sensitivity^37–39^. However, several negative effects are associated with chronic calorie restriction regimens^36,40,41^, such as increased hunger, reduced body temperature leading to a feeling of being cold, malnutrition and body weight loss that would not be recommendable for the early or late stages of life. In this study, we removed the constant hypocaloric component in a protocol of daytime restricted feeding in rats, extending the time of access to food from 2 to 5 h, using standard lab rodent diet, and after 21 days of each protocol, we analysed the effect on behavioural, metabolic, and clock parameters in NCT compared to HCT. The kinetic of food intake in the 5 h food access group displayed a process of adaptation that led to eat equivalent calories to AL in the third week. We made sure that the hypocaloric condition in HCT did not entail malnutrition. Accordingly, serum proteins and circulating lactate dehydrogenase activity were normal in the HCT group despite the calorie restriction^23,42^, could be explained as being part of the rheostatic adjustments promoted by TRF^43^.

Certainly, several timekeeping mechanisms are implicated in the FAA, which occurs before mealtime when food is available only for a few hours per day. These mechanisms include neuronal activation, molecular clock entrainment, hormonal cues, and metabolic regulation involving the redox state^44,45^. Previous studies demonstrated that hypocaloric but not normocaloric TRF induces a phase advance of locomotor activity rhythm in mice^46^. In our study, rats under NCT displayed FAA in a similar pattern and intensity to that of rats under HCT (Fig. 2), indicating that achieving normocaloric intake induces this behavioural hallmark of the FEO.

**Figure 7:**
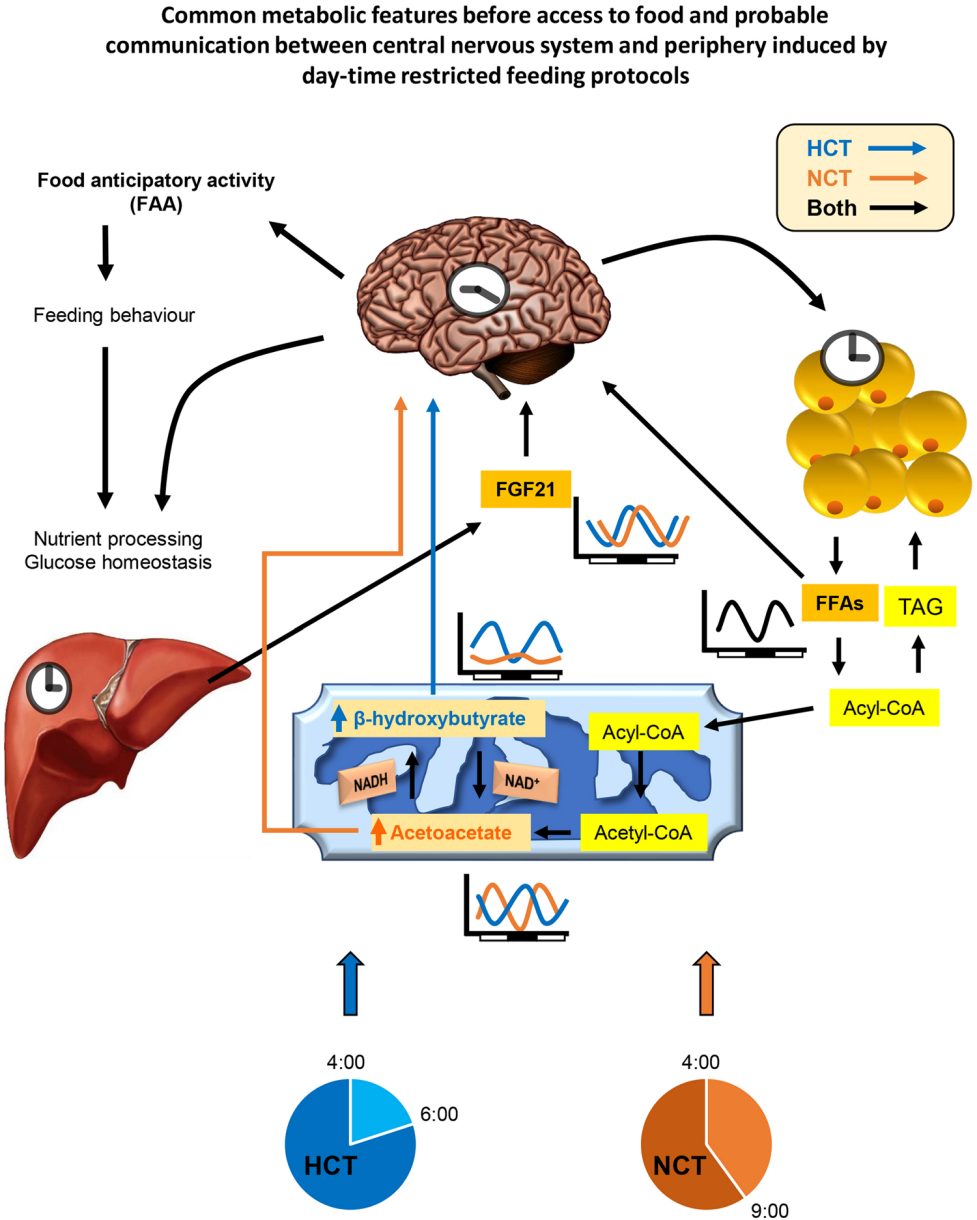
Schematic representation of the effects of HCT and NCT over metabolic homeostasis. Metabolic functions, peripheral clocks, and behaviour are regulated by the feeding. Ketone bodies and free fatty acids (FFAs) as energy sources and signaling molecules. FGF21 as a regulator of metabolism and circadian behaviour. Regardless of the calorie intake and lipid storage, adipose tissue in both TRF conditions performs lipolysis to induce a similar food anticipatory activity (FAA) response. Arrows colour indicates pathways induced by HCT (blue, food access ZT4-ZT6), NCT (orange, food access ZT4-ZT9) or both (black). Triacylglycerol (TAG).

The link between peripheral organs and the central nervous system to induce the FAA is still under study. Until now, it is known that synchronization of food entrainable clock is driven by a complex multilayer mechanism that involves a coordinated wide variety of regulatory pathways, such as humoral, enzymatic, and redox state in peripheral and cerebral structures^45^. Some metabolites have been proposed as candidates to perform this communication. β-hydroxybutyrate is a ketone body synthesized by the liver from fatty acids in response to fasting or a low-carbohydrate condition. Aside from its role as an energy source, β-hydroxybutyrate has cellular signalling actions^47^. In this regard, β-hydroxybutyrate participates in FAA modulation, and its hepatic production is regulated by the clock protein Per2^48^. In addition to these findings, we observed that β-hydroxybutyrate increased in serum before the time of food access, only in HCT, whereas acetoacetate was prominently enhanced under NCT. Under sustained hypocaloric conditions, both ketone bodies displayed a clear oscillation between oxidised, neutral, and reduced states, while under NCT, they displayed a defined oxidised state, suggesting a differential mitochondrial redox adaptation depending on the calorie intake. Thereby, other feeding cues induced in the absence of a continuous calorie restriction could be participating in a complex mechanism as signaling molecules in rising FAA, such as acetoacetate^49^, fatty acids^50,51^, and FGF21^31,52,53^. Even though Fgf21 knockout mice display FAA under TRF^54^, it is clear that both, HCT and NCT induced a shift phase of the FGF21 rhythmic expression in the liver, thus we suggest that FGF21 is dispensable but necessary when is present, as part of a whole in a coordinated response from peripheral to neural centres. The metabolic state can induce sensitisation in several areas of the brain, mainly hypothalamic and hippocampal neurons, to coordinate neuroendocrine and metabolic effectors of energy balance^49,51,55,56^. In response to fasting, enhanced ketone bodies, FFAs, and FGF21 in both groups of TRF, displaying either a phase shift in their rhythmic pattern or a significant peak before feeding time (Figs. 5I–K and 6A–C), could signal to diverse brain areas implicated in the timing of food anticipation, thus favouring food-seeking behaviour (Fig. 7). One of the most distinctive metabolic adaptations associated with HCT is the peak of circulating FFAs during the display of the FAA (at ZT3)^10^. Interestingly, even with different calorie intake, we observed a similar response under NCT that indicates lipolytic activation of the adipose tissue and has several implications. Besides serving as energy sources in different tissues, FFAs may be utilised in the liver as substrates for peroxisomal and mitochondrial oxidation to generate metabolic energy (ATP supply) and sustain ketogenesis to release of energetic substrates into the bloodstream^57^. Moreover, fatty acids can function as ligands of G protein-coupled receptors and act as ligands for nuclear receptor transcription factors involved in energetic metabolism such as peroxisome proliferator-activated receptors or PPAR^58^. In this regard, PPARα is critical for the fasting response; its daily rhythmicity is modified by HCT^10^, and its positive regulator PGC1-α, a key regulator of energy homeostasis, is highly expressed in HCT and induced by FGF21^9,29^. As well, PPARα activation positively regulates the expression of FGF21^59^. FGF21 activates lipolysis, and even the differences in size and fat content, similarly in both groups by gonadal and perirenal adipose tissue to release FFAs for oxidative metabolism. It has been reported that FGF21 administration induces sympathetic nerve activity in brown adipose tissue to affect energy expenditure^53^, and contributes to the control of white adipocyte lipolysis^60^, suggesting a similar mechanism of action in white adipose tissue under HCT and NCT.

In humans, nutritional regimens that involve restricting energy intakes, such as intermittent fasting or calorie restriction, have demonstrated their effectiveness in favouring weight loss and improving diabetogenic parameters like visceral fat mass and fasting insulin. However, their effect on lowering glucose levels is still controversial and under investigation^61^. Regarding the effects on the daily average glycaemia, we observed a hypoglycaemic effect under NCT and HCT since the beginning of the experiment, probably due to the hypocaloric intake in the first week. Average glycaemia remains similar in both HCT and NCT, which could be due to the effect that peripheral metabolic signals (FFAs) have on glucose homeostasis by regulating hepatic gluconeogenesis and glycogenolysis through central actions^51,62^. A similar glycaemic response is elicited despite different dynamics of circulating insulin, hepatic glycogen, and serum corticosteroid. This response favours hepatic gluconeogenesis with a different circadian dynamic. The expression of gluconeogenic enzymes G6Pase and PEPCK gene underwent a phase shift, with the acrophase during the night phase in HCT and before feeding in NCT. Importantly, the blood glucose peak accompanied by the blood insulin peak seen in HCT may be detrimental to long-term glucose homeostasis, but the maintenance of low blood glucose levels throughout the day, with the discrete insulin response, as seen in NCT, may represent a way to prevent or control pathologies such as metabolic syndrome and diabetes.

Regarding the pro-oxidant status of the liver, the lower values of conjugated dienes in NCT are indicative of reduced pro-oxidant driving compared to AL, despite the similar food calorie input at the end of the protocol. In contrast, HCT elicited a noticeable 24 h rhythm in liver-conjugated dienes, suggesting that the pro-oxidant reactions in this group are chronobiologically regulated by sustained hypocaloric intake. Because the data obtained in the TBARs assay were different from the conjugated dienes results, it can be postulated that the onset and propagation of the pro-oxidant reactions in each of the studied groups are differentially controlled. These results suggest that both food synchronization protocols differentially modulate the pro-oxidant status of the liver.

Metabolic homeostasis is maintained, in part, by mutual regulation between components of the circadian clock and metabolic networks^63^. Interestingly, basal metabolism, visualised as energy expenditure and respiratory ratio quotient, showed high resemblance in HCT and NCT, consistent with a similar response in FGF21 and FFAs. Both restricted feeding protocols had a global entrainment effect on the liver clock as shown by the similar phase shift of Per1 and the close phase shift of Bmal1 and Rev-Erb-α, which magnitude was affected by calorie intake. Thus, overall changes in metabolism handling under HCT and NCT result in a similar metabolic state supplied by the synchronizing effect of timely restricted food access, regardless of the calorie intake, with similarities and differences in the metabolic dynamic.

In summary, our data indicate that sustained hypocaloric and progressive achievement of normocaloric timely food restriction activate lipolysis in the adipose tissue to elicit a peak of FFAs during the FAA. However, the hepatic processing of fatty acids differed according to the caloric intake. Entrainment in clock gene expression in the liver could put in phase transcriptional and metabolic activities to differentially coordinate local responses and a global metabolic adaptation as part of the rheostatic adjustments associated with FEO establishment^43^. TRF with progressive increasing calorie intake appears to be effective in keeping synchronization and metabolic profits. Finding a regimen that promotes satiety and abates hunger may be an option for people adhering to a diet. Thus, restricted feeding schedules with a progressive increase of calories to achieve normocaloric intake could be a good option to improve metabolic parameters and likely control body weight.

## Methods

### Experimental animals

All animal experiments were carried out with approval from the Institutional Animal Care and Use Committee at the Institute of Neurobiology, Universidad Nacional Autónoma de México (UNAM). Rats were handled in accordance with the Institutional Guide for the Care and Use of Animals under Biomedical Experimentation at Universidad Nacional Autónoma de México (UNAM) and standards for medical biological rhythm research^64^.

Adult male Wistar rats were used for the experiments. At the beginning, rats weighed 240 ± 10 g and were maintained under 12:12 h light-dark cycles (lights on at 08:00 h and off at 20:00 h) and at constant temperature (22 ± 1 °C). Rats were individually housed to record their activity or kept in groups of 4 in transparent acrylic cages (40 ×50 ×20 cm) for metabolic and molecular analysis. All groups were fed with normal chow (3.02 kcal/g, 5001 Rodent diet, LabDiet, St. Louis, MO) and water *ad libitum* for 1 week of habituation. Afterwards, rats were randomly assigned to time-restricted feeding groups (weight 300 ± 15 g).

### Experimental design

Rats were randomly assigned to one of 3 groups: 1) rats fed *ad libitum* (AL); 2) progressive increasing of calorie intake to accomplish normocaloric time-restricted feeding (NCT), in which rats were fed for 5 h (ZT4–ZT9) every day; and 3) sustained hypocaloric time-restricted feeding (HCT), in which rats were fed for 2 h (ZT4-ZT6) every day (Fig. 1A). After 3 weeks, rats were euthanized by decapitation every 3 h in a 24 h day-night cycle at ZT: 0, 3, 6, 9, 12, 15, 18, and 21. The feeding protocols were performed with a minimum of 4 rats and repeated in 3 independent batches of rats. Additionally, groups of 4 rats were: (1) fasted for 21 h, (2) fasted for 21 h and refed for 2 h, (3) fasted for 21 h and refed for 5 h, and euthanized immediately after fasting (ZT3) or refeeding (ZT6 and ZT9, respectively).

### Food intake and body weight measurements

Body weight was determined for all groups once a week at ZT2. Food intake was recorded at ZT2 every day for the AL group and at the end of the access to food for the NCT and HCT groups (ZT6 and ZT9, respectively). Data were analysed according to the Hanes–Woolf method^65^ to linearize the saturating curves of food intake.

### Metabolic cages and nutritional markers

The metabolic performance was analysed individually in metabolic cages by indirect calorimetry with an OxyletPro System (Panlab Harvard Apparatus, Barcelona, Spain) according to the manufacturer’s instructions and data analysis were performed with Metabolism V 3.0 software (Panlab, Harvard Apparatus, Barcelona, Spain) (https://www.harvardapparatus.com/oxyletpro-system-physiocage-1.html). For the analysis, 2 rats under AL, HCT, and NCT were used and metabolic parameters were measured every 20 min for 3 days following 2 days of habituation. Photoperiod and feeding conditions were kept the same as in the home cages. As a marker of nutritional status, total protein was measured in serum by the Bradford method. Lactate dehydrogenase activity was determined by an enzyme-coupled reaction to the reduction of NAD^+^ to NADH using the conversion of lactate to pyruvate measured at λ = 340 nm.

### Locomotor activity recordings

Locomotor activity in rats was recorded employing a motion detection system based on an infrared light-detecting crossing and data collected by ACTIBIO V 1.0 software (developed by Network, Computing, and Software Development Unit, URIDES, of School of Psychology, UNAM.)^66^. After 1 week of acclimation to the activity recording system, rats with food available AL were monitored for 1 week and then, randomly grouped to be subjected either to HCT or NCT for 3 weeks. Each rat was monitored individually inside an acrylic cage. Room environment was maintained between 23 and 25 °C and light-dark cycles 12:12 h (photo-phase 08:00–20:00 h; 200–250 lux). Ventilation inside the chamber was kept constant. To evaluate the persistence of the FAA after 3 weeks of restricted feeding, 3 rats were subjected to constant darkness and fasting conditions for two days. Individual locomotion profiles were calculated using the number of infrared light crossings recorded by a computer every 10 min (bin-cell). Each beam interruption was considered as a single event. Locomotor activity data were analysed through double plotted actograms, and average waveforms by using Actiview V 1.0 software (Minimitter, Sunriver, OR); (https://www.jdinstruments.com/vsoftware.html). Locomotor activity was recorded in 2 groups of rats (n = 10). First, rats were kept for 10 days in AL feeding conditions in LD. Then, time-restricted feeding was followed for 21 days to establish 5 h or 2 h of daily food access, as described above.

### Somatometry and histology analysis

After 21 days of each feeding protocol, nutrient-responsive organs (stomach, liver, perigonadal and perirenal adipose tissue) were removed from the rats that were euthanized every 3 h in a 24 h day-night cycle, as described above. The organs were quickly weighed and immediately frozen in dry ice or fixed in 10% buffered formaldehyde solution, embedded in paraffin and cut into 7 µm slices. Liver and adipose tissues from the rats under HCT, NCT and a 21 h fasting (Fa), euthanized before (ZT3) or after food intake (ZT6 for HCT or ZT9 for NCT), were stained with a standard hematoxylin-eosin (H&E) method. A total of 68 high-resolution images (24-bit colour) in TIFF format were obtained from the adipose tissues of 4 rats and captured (Leica ICC50 HD camera) at a resolution of 2048 ×1536 pixels using LAS EZ V 3.0 software (Leica Microsystems, Heerbrugg Switzerland) (https://www.leica-microsystems.com/es/productos/software-de-microscopia/detalles/product/show/Products/leica-las-ez/). These images of 1,200 to 1,400 adipocytes per experimental group were processed with Adiposoft V 1.15 software (developed at the Imaging Unit of the Center for Applied Medical Research (CIMA), University of Navarra)^67^ (https://imagej.net/Adiposoft). Images were obtained from the liver samples of 4 rats, and 60 hepatocytes around the central vein per sample were analysed with ImageJ software (National Institutes of Health, Bethesda, MD, USA)^68^.

### Analysis of mRNA expression

Daily profile of mRNA expression was analysed as we previously described^69^ with little modifications. Briefly, after 21 days of each feeding protocol, 20–30 mg of liver tissue were collected every 3 h throughout the 24 h day-night cycle. Total RNA was isolated using the SV Total RNA Isolation System (Promega, Madison, WI, USA). RNA (2 μg) was reverse transcribed using a RevertAid First Strand cDNA Synthesis Kit (Thermo Scientific, Waltham, MA, USA). RT and qPCR were performed using the CFX96 real-time PCR detection system (Bio-Rad, Hercules, CA, USA). Primers used for qPCR amplification were synthesized by Sigma-Aldrich Co. (St. Louis, MO, USA); the corresponding sequences and annealing temperatures are listed in Supplementary Table S1. Amplifications were carried out in a 10 μl final reaction volume containing cDNA diluted 1/100 and 0.5 μM of each of the primer pairs in Maxima SYBR Green qPCR Master Mix (Thermo Fisher Scientific, Waltham, MA, USA). Data were analysed by the 2^−ΔΔC^_T_ method and cycle thresholds (CT) were normalised to the Rps18 mRNA expression used as housekeeping gene.

### Blood glucose, insulin, corticosterone, FFAs, FGF21, and liver glycogen measurements

Every week, 4 rats of each experimental group (HCT, NCT, and AL) were randomly selected for measurements. Blood glucose obtained by tail puncture was measured every 3 h throughout the day. For the GTT, 5 rats randomly selected from each group were fasted (fasting started at ZT0 for AL, at ZT4 for HCT and at ZT9 for NCT) and after 8 h, tail blood was collected (time point 0). Immediately, 2 g/kg of glucose per body weight were injected intraperitoneally, and tail blood was collected 15, 30, 60, 120, and 180 min after for glucose determination. In both procedures, a glucometer (Contour TS, Bayer, USA) was used. At the end of the experiment, blood obtained every 3 h in a 24 h cycle was centrifuged to recuperate serum fraction, and hormones were quantified using ELISA assays for insulin (ALPCO Diagnostics, Windham, NH, USA), corticosterone (R&D Systems, Minneapolis, MN, USA), and FGF21 (Elabscience Biotechnology Co., Ltd, Houston, TX, USA) according to the manufacturer’s instructions. FFAs were quantified using a colourimetric kit (Sigma-Aldrich, St. Louis, MO, USA). Glycogen was measured in paraffin-embedded liver tissues from 4 rats every 3 h throughout a day profile in the NCT group and before and after food access in the HCT group by periodic acid–Schiff (PAS) staining. Images were taken with a digital camera (Olympus DP71) and staining was quantified with ImageJ software.

### Determination of ketone bodies, mitochondrial redox state, and lipid peroxidation

After 21 days of each feeding protocol, liver pro-oxidant reactions were measured in terms of conjugated dienes and TBARs. Conjugated dienes were quantified in liver homogenates. The lipidic fraction was recuperated with Folch reactive (chloroform-methanol 2:1). Samples were allowed to air-dry and then solubilised in hexane to be measured at λ = 233 nm. TBARs were measured in liver homogenates as previously reported^70^.

Ketone bodies were measured in serum of rats after 21 days of HCT or NCT protocols by biochemical assays^71,72^. As control groups, we used a 21 h of fasting and fasting followed by refeeding for 2 h. Serum was treated with 6% perchloric acid and neutralised with 5 mol/L of K_2_CO_3_ and used for metabolite determinations by enzymatic methods: β-hydroxybutyrate and acetoacetate. For measuring β-hydroxybutyrate, deproteinized samples reacted with NAD^+^ at pH 8.5 to oxidize β-hydroxybutyrate to acetoacetate, which was removed in the form of its hydrazone to avoid to promote a reversible reaction, in the presence of β-hydroxybutyrate dehydrogenase (Sigma-Aldrich, St. Louis, MO, USA). The increase of absorbance at λ = 340 nm due to the formation of NADH was a measure of the reaction. For measuring acetoacetate, deproteinized samples reacted with an excess of NADH at pH 7.0 to reduce acetoacetate to β-hydroxybutyrate in the presence of β-hydroxybutyrate dehydrogenase. The decrease of absorbance at λ = 340 nm due to the formation of NAD was measured. The mitochondrial NAD^+^/NADH ratio was estimated using the following equation: NAD^+^/NADH = [oxidised substrate]/[reduced substrate] × 1/*K*_*eq*_ (*K*_*eq*_ of β-hydroxybutyrate dehydrogenase = 4.93 × 10^−2^ M). The mitochondrial redox state was calculated from the β-hydroxybutyrate/acetoacetate ratio, in accordance with Stubbs *et al*.^73^.

### Statistical analysis

Results were expressed as mean ± SEM. Data were replicated in two independent experiments and with 4–12 rats for each sampling time. The statistical analyses were performed and all graphics were created using Sigma Plot v 12.0 software (Systat Software Inc., San José, CA, USA) (https://systatsoftware.com/products/sigmaplot/). Normality distribution was determined by the Shapiro-Wilk test. Data showing a parametric distribution or equal variances, significant differences between groups were determined by a two-way ANOVA with a post-hoc Tukey comparison test (see Supplementary Table S3). Statistical differences between different points and between temporal points in the time curves were determined by a one-way ANOVA followed by a post-hoc Tukey comparison test. All pairwise multiple comparisons were performed with a Student’s t-test (see Supplementary Table S4). Data that showed a non-parametric distribution were determined by a Kruskal–Wallis one-way ANOVA with a Dunn’s post-hoc test. Pairwise multiple comparisons were performed using a Mann–Whitney U test. Differences in means with *P* < 0.05 were considered statistically significant. Chronobiological analysis was performed by COSANA V 1.0 software (Beneditto-Silva; GMDRB, Department of Fisiologia e Biofisica, ICB/University of Sao Paulo, Br.)^74^.

### Supplementary information

Supplementary information.
