# Author Correction: Synchronization of the circadian clock by time-restricted feeding with progressive increasing calorie intake. Resemblances and differences regarding a sustained hypocaloric restriction

**DOI:** 10.1038/s41598-024-66718-2

**Published:** 2024-07-10

**Authors:** Ana Cristina García-Gaytán, Manuel Miranda-Anaya, Isaías Turrubiate, Leonardo López-De Portugal, Guadalupe Nayeli Bocanegra-Botello, Amairani López-Islas, Mauricio Díaz-Muñoz, Isabel Méndez

**Affiliations:** 1 Instituto de Neurobiología, Universidad Nacional Autónoma de México (UNAM), Campus UNAM-Juriquilla, 76230 Querétaro, Mexico; 2 Unidad Multidisciplinaria de Docencia e Investigación, Facultad de Ciencias, Universidad Nacional Autónoma de México (UNAM), Campus UNAM-Juriquilla, 76230 Querétaro, Mexico

Correction to: *Scientific Reports* 10.1038/s41598-020-66538-0, published online 22 June 2020

The original version of this Article contained an error in the Acknowledgements section.

 “We thank Daisy Gasca Martínez for the technical advice for metabolic cages and specialised software and Fernando López, Olivia Vázquez-Martínez, Ma. de Lourdes Lara, Alejandra Castilla, and Martín García for their technical assistance. We thank Summer Science Program students Darío Sandoval Valdez, Itzel Ramos Rodríguez, Perla Velázquez Pérez, for their technical assistance. Ana Cristina García-Gaytán is a Doctoral student from the Programa de Posgrado en Ciencias Biomédicas, Universidad Nacional Autónoma de México (UNAM) and received fellowships from Mexico’s National Council of Science and Technology (CONACyT). We thank Jessica Norris for critically editing the manuscript. This study was supported by DGAPA-PAPIIT, UNAM México (IN202515), DGAPA-PAPIIT, UNAM México (IN200815) and CONACyT, México (284–557).”

now reads:

“We thank Daisy Gasca Martínez for the technical advice for metabolic cages and specialised software and Fernando López, Olivia Vázquez-Martínez, Ma. de Lourdes Lara, Alejandra Castilla, and Martín García for their technical assistance. We thank Summer Science Program students Darío Sandoval Valdez, Itzel Ramos Rodríguez, Perla Velázquez Pérez, for their technical assistance. Ana Cristina García-Gaytán is a doctoral student from the Programa de Doctorado en Ciencias Biomédicas, Universidad Nacional Autónoma de México (UNAM) and has received CONAHCYT fellowship #239250. We thank Jessica Norris for critically editing the manuscript. This study was supported by DGAPA-PAPIIT, UNAM México (IN202515), DGAPA-PAPIIT, UNAM México (IN200815) and CONACyT, México (284–557).”

The original Article has been corrected.

