# Supplementary material for: Synchronization of the circadian clock by time-restricted feeding with progressive increasing calorie intake. Resemblances and differences regarding a sustained hypocaloric restriction
Source: Sci Rep. 2020 Jun 22;10:10036. doi: 10.1038/s41598-020-66538-0 (PMC7308331; doi:10.1038/s41598-020-66538-0)
Supplement: Supplementary file 1 — Supplementary information. [file 41598_2020_66538_MOESM1_ESM.pdf]

Synchronization of the circadian clock by time restricted feeding with progressive increasing calorie intake. Resemblances and differences regarding a sustained hypocaloric restriction

Ana Cristina García-Gaytán[1,+], Manuel Miranda-Anaya[2,+], Isaías Turrubiate[1,+], Leonardo López-De Portugal[1], Guadalupe Nayeli Bocanegra-Botello[1], Amairani López-Islas[1], Mauricio Díaz-Muñoz[1], Isabel Méndez[1,*].

[1]Instituto de Neurobiología, Universidad Nacional Autónoma de México (UNAM), Campus UNAM-Juriquilla. Querétaro, 76230, México.

[2]Unidad Multidisciplinaria de Docencia e Investigación, Facultad de Ciencias, Universidad Nacional Autónoma de México (UNAM), Campus UNAM-Juriquilla. Querétaro, 76230, México.

[+] contributed equally to this work.

* corresponding author (isabelcm@unam.mx)

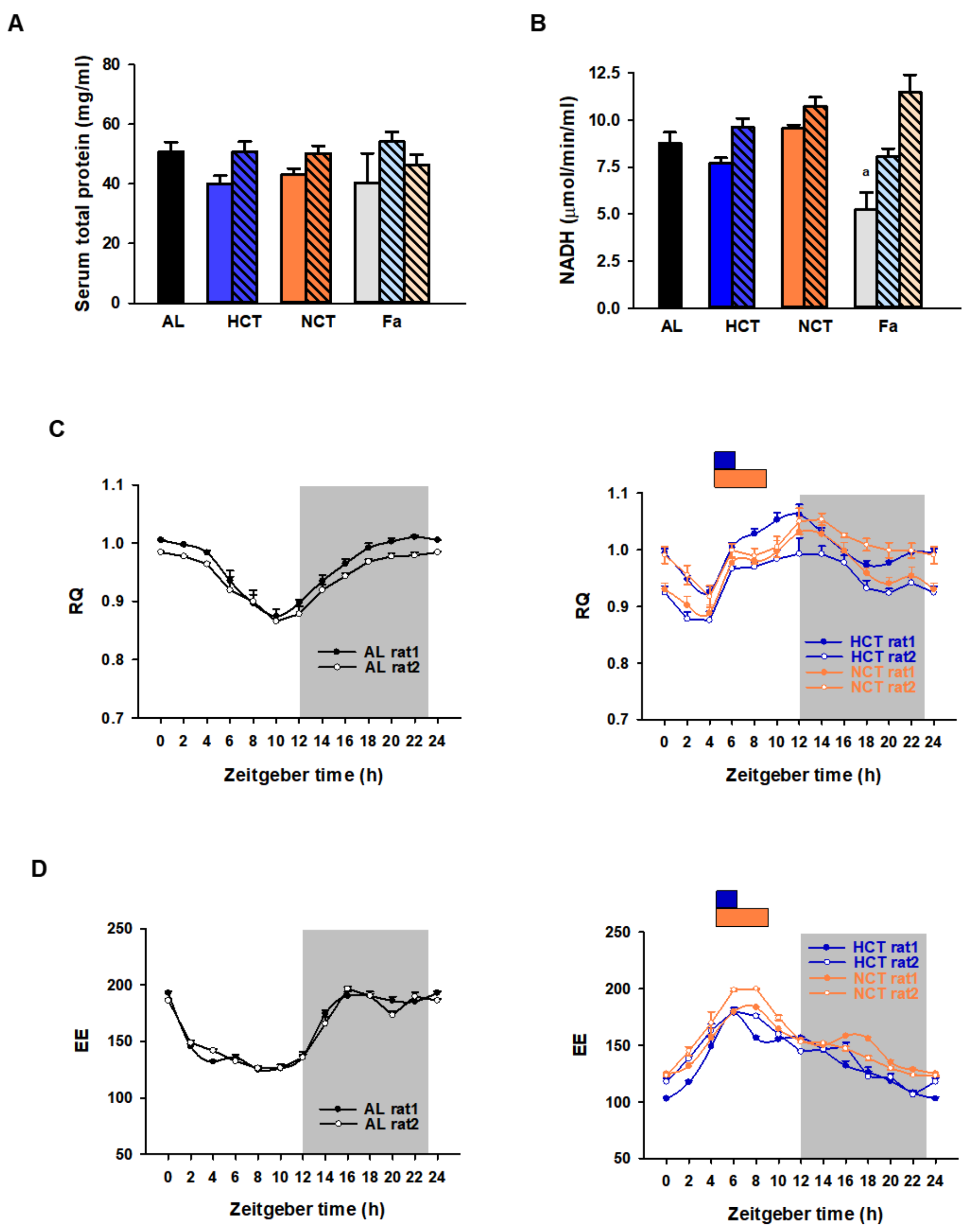

Supplementary Figure 1. Nutritional and metabolic status under AL, HCT, and NCT. (A) Total protein and (B) lactate dehydrogenase in serum as markers of nutritional status were evaluated before (ZT3) and after feeding (ZT6 in HCT, ZT9 in NCT, dashed bars) after 21 days of each time restricted feeding protocol. A single 21 h fasting at ZT3 (Fa, grey) and dashed bars represent refeeding for 2 h (clear blue, ZT6) and 5 h (clear orange, ZT9). *P*<0.05 a vs AL. (C) 24 h respiratory quotient (RQ) (ratio CO_2 produced/O_2 consumed) in AL (left) and HCT and NCT (right). (D) 24 h energy expenditure (EE) in AL (left) and HCT and NCT (right). Each point represents the average of recording for 3 days.

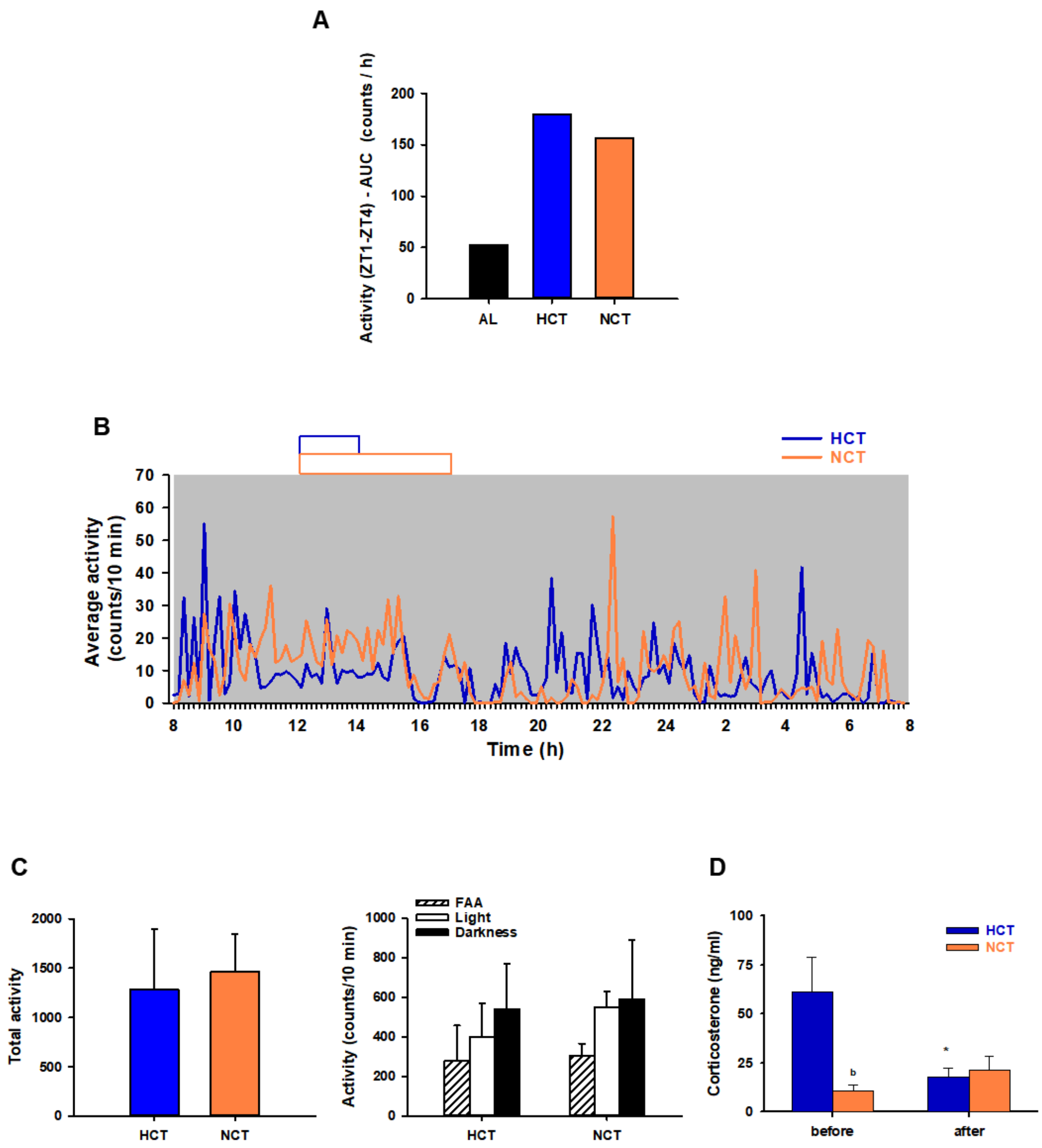

Supplementary Figure 2. Food anticipatory activity and persistence. (A) The area under the curve 3 h before food access (ZT1-ZT4) corresponding to spontaneous basal activity under AL condition and the food-anticipatory activity (FAA) under HCT and NCT in 12:12 h light-dark cycles. (B-C) Locomotor activity in constant conditions: fasting and 12:12 h dark-dark conditions over a 24 h cycle, after 21 days of HCT and NCT. (B) Activity profiles of rats. Waveforms represent the sum into 10-min bins and the average of a one-day recording. Horizontal bars represent the expected time of food access defined in the previous light-dark cycles (blue, HCT; orange, NCT). Each point represents the recording average of 3 rats. (C-left) Average of the total activity in all groups; (C-center) Representation of activity in circadian time during FAA (9:00-12:00 h), the rest of the “light period” (12:00-20:00 h) and “darkness” (20:00-8:00 h). (D) Corticosterone levels measured before and after food access in NCT and HCT (n= 4 rats). *P*<0.05 b vs HCT, * vs before food access in their respective group.

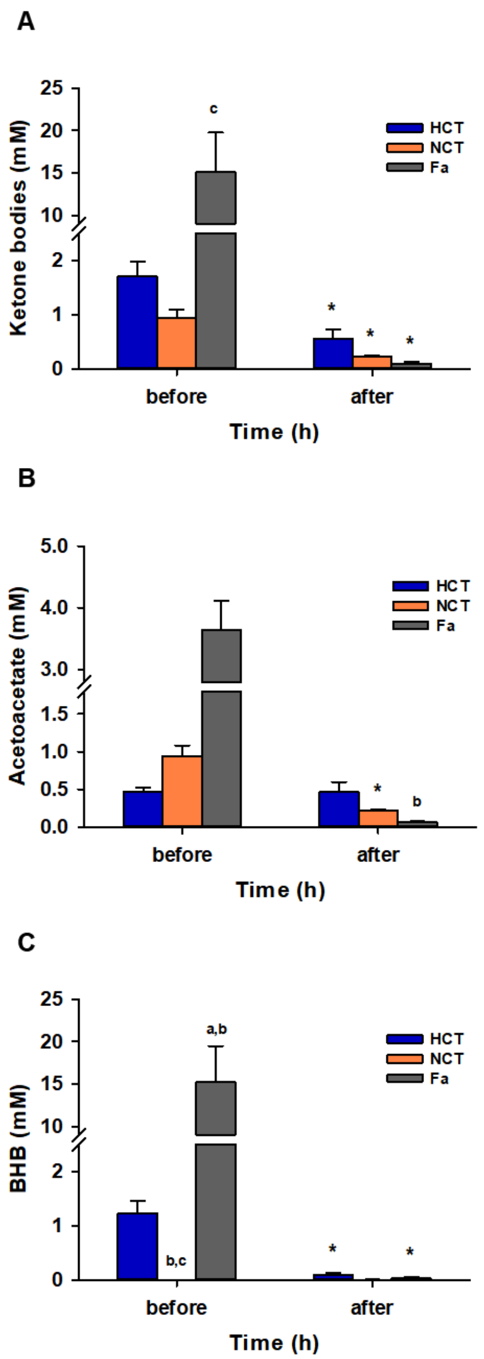

Supplementary Figure 3. Effect of fasting (Fa), refeeding, HCT and NCT (n= 4 rats) on ketone bodies. Serum ketone bodies measured before (ZT3) and after feeding (ZT6 in HCT or Fa, ZT 9 in NCT) after 21 days of each time restricted feeding protocol. A single 21 h fasting at ZT3 (Fa, grey) and dashed bars represent refeeding for 2 h (clear blue, ZT6) and 5 h (clear orange, ZT9). (A) Total ketone bodies, (B) acetoacetate and (C) β-hydroxybutyrate (BHB). Bars represent the mean ± SEM. P<0.01 a vs AL, b vs HCT, c vs NCT. P<0.05 * vs before food access in their respective group.

Supplementary Table S1. Oligonucleotides used as qPCR primers

Gene	Oligonucleotide sense	Oligonucleotide antisense	Amplicon length (bp)	Annealing temperature (°C)	NCBI reference
**Arntl**	CTGCCTCGTTGCAATCGGGC	GCCAAAATAGCCGTCGCCCTCT	149	65	NM_024362.2
**Per1**	AGCCCCCGGAGTCGGACATC	GGGACAGCACGGCCTTGGTC	149	68	NM_001034125.1
**Nr1d1**	CTTCCGTGACCTTTCTCAGC	CTGGTCCTTCACGTTGAACA	82	63.7	NM_145775.2
**G6pc**	GATCGCTGACCTCAGGAACGC	AGAGGCACGGAGCTGTTGCTG	197	70	NM_176077.3
**Pck1**	ACGGTGGGAACTCACTGCTTG	TGCCTTCGGGGTTAGTTATGC	122	64.5	NM_198780.3
**Fgf21**	CAGGGGTCATTCAAATCCTG	CAAAGTGAGGCGATCCATAG	87	59	NM_130752.1
**Rps18**	TTCAGCACATCCTGCGAGTA	GCAGACATTGACCTCACCAA	115	62	NM_213557.1

Supplementary Table S2. Feeding parameters according to Hanes-Woolf analysis

Group	Food intake$_{max}$/2 (days)	Food intake$_{max}$ (kcal)	Food intake$_{max}$ (%)
AL	0.25	127.4	100
HCT	2.78	76.8	60
NCT	2.65	118.6	93

Supplementary Table S3. Values for two-way ANOVA for several temporal parameters measured during restricted feeding protocols (food intake and body mass and glucose) or at the end of the protocols (day 21); ns, non significant.

Parameter	Feeding condition		Time		Interaction	
	F	*P*	*F*	*P*	*F*	*P*
**Food intake**	$F_{(2,117)}$ = 249.541	**<0.001**	$F_{(7,117)}$ = 8.847	**<0.001**	$F_{(14,117)}$ = 1.236	ns
**Body mass**	$F_{(2,30)}$ = 33.793	**<0.001**	$F_{(4,30)}$ = 40.599	**<0.001**	$F_{(8,30)}$ = 6.982	**<0.001**
**Arntl mRNA**	$F_{(2,72)}$ = 9.35	**<0.001**	$F_{(7,72)}$ = 2.214	**<0.05**	$F_{(14,72)}$ = 2.214	**<0.001**
**Per1 mRNA**	$F_{(2,72)}$ = 1.982	ns	$F_{(7,72)}$ = 9.721	**<0.001**	$F_{(14,72)}$ = 6.848	**<0.001**
**Nrd1 mRNA**	$F_{(2,72)}$ = 5.253	**<0.001**	$F_{(7,72)}$ = 4.268	**<0.05**	$F_{(14,72)}$ = 7.773	**<0.001**
**G6pc mRNA**	$F_{(2,72)}$ = 6.094	**<0.01**	$F_{(7,72)}$ = 4.013	**<0.001**	$F_{(14,72)}$ = 9.805	**<0.001**
**Pck1 mRNA**	$F_{(2,71)}$ = 0.819	ns	$F_{(7,71)}$ = 1.604	ns	$F_{(14,71)}$ = 7.658	**<0.001**
**Fgf21 mRNA**	$F_{(2,71)}$ = 0.126	ns	$F_{(7,71)}$ = 1.309	ns	$F_{(14,71)}$ = 7.27	**<0.001**
**Glucose (week 1)**	$F_{(1,48)}$ = 4.108	**<0.05**	$F_{(7,48)}$ = 5.09	**<0.001**	$F_{(7,48)}$ = 0.876	ns
**Glucose (week 2)**	$F_{(1,48)}$ = 0.163	ns	$F_{(7,48)}$ = 2.705	**<0.05**	$F_{(7,48)}$ = 0.603	ns
**Glucose (week 3)**	$F_{(2,96)}$ = 0.00405	ns	$F_{(7,96)}$ = 2.511	**<0.05**	$F_{(14,96)}$ = 1.695	ns
**GTT**	$F_{(2,48)}$ = 6.305	**<0.01**	$F_{(5,48)}$ = 60.783	**<0.001**	$F_{(10,48)}$ = 2.129	**<0.05**
**Total ketone bodies**	$F_{(1,47)}$ = 121.534	**<0.001**	$F_{(7,47)}$ = 12.432	**<0.001**	$F_{(7,47)}$ = 9.308	**<0.001**
**Acetoacetate**	$F_{(1,44)}$ = 27.902	**<0.001**	$F_{(7,44)}$ = 3.185	**<0.01**	$F_{(7,44)}$ = 5.75	**<0.001**
**Beta-hydroxybutyrate**	$F_{(1,47)}$ = 55.815	**<0.001**	$F_{(7,47)}$ = 5.944	**<0.001**	$F_{(7,47)}$ = 6.524	**<0.001**
**Conjugated dienes**	$F_{(2,72)}$ = 22.575	**<0.001**	$F_{(7,72)}$ = 146.946	**<0.001**	$F_{(14,72)}$ = 18.641	**<0.001**
**TBARS**	$F_{(2,67)}$ = 31.404	**<0.001**	$F_{(7,67)}$ = 10.361	**<0.001**	$F_{(14,67)}$ = 9.863	**<0.001**
**TBARS + Fe^{2+}**	$F_{(2,68)}$ = 52.809	**<0.001**	$F_{(7,68)}$ = 6.319	**<0.001**	$F_{(14,68)}$ = 2.933	**0.002**

Supplementary Table S4. Values for Student's t-test for several parameters measured at the end (day 21) of the respective restricted feeding protocol; ns, non significant

Parameter	Feeding condition	
	t	*P*
**Acetoacetate**	2.22	ns
**Beta-hydroxybutyrate**	3.019	**<0.01**
**Stomach weight**	8.791	**<0.01**
**Insulin**	4.037	**<0.01**
**Perirenal adipose tissue weight**	26.822	**<0.001**
**Corticosterone**	-3.399	**<0.05**
